# Affordable Arsenic Detection Device Using Handmade Lokta Paper for Decentralized and Sustainable Manufacturing

**DOI:** 10.1155/jamc/9918530

**Published:** 2026-05-23

**Authors:** Ramdeep Shrestha, Bhanu Bhakta Neupane, Basant Giri

**Affiliations:** ^1^ Central Department of Chemistry, Tribhuvan University, Kirtipur, Nepal, tribhuvan-university.edu.np; ^2^ Center for Analytical Sciences, Kathmandu Institute of Applied Sciences, P. O. Box 23002, Kathmandu, Nepal

**Keywords:** arsenic contamination, *Daphne*, paper strip, traditional material

## Abstract

The reliance on centralized manufacturing systems often shows vulnerability of global supply chains, as evidenced by disruptions during the COVID‐19 pandemic and geopolitical conflicts. This study reports an affordable and portable paper strip sensor for arsenic detection in groundwater, developed using distributed manufacturing and sustainability principles. Handmade Lokta Paper (HLP), produced in the laboratory from bark of a native Lokta plant, *Daphne*, through eco‐friendly methods, served as the sensor substrate. The HLP strip employed the Gutzeit reaction and integrated smartphone imaging for qualitative, semiquantitative, and quantitative arsenic analyses. The assay optimization was achieved with 1% (w/v) HgBr_2_ impregnation, 5‐min dipping time, 15‐min arsine exposure, and imaging within 25 min. Testing of 21 groundwater samples from Nepal revealed strong agreement between the HLP strip and a commercial kit Econo‐Quick (EQ) (Cohen’s kappa = 0.811). The HLP strip demonstrated a lower limit of detection (18 vs. 20 µg/L) and limit of quantification (54 vs. 61 µg/L). Most water samples had arsenic concentrations below 10 µg/L, with few reaching Nepal’s regulatory limit of 50 µg/L. In addition to robust analytical performance, the HLP strip exemplifies the scalability and cost‐effectiveness of arsenic sensors, with a production cost of USD 0.37 per test—significantly lower than commercial alternatives. This innovation may foster local economic growth, minimize supply chain dependency, and reduce environmental impact through the sustainable utilization of Lokta paper. The study highlights the potential of integrating traditional materials with advanced analytical techniques to address global health and environmental challenges while promoting regional resilience and sustainability.

## 1. Introduction

The large‐scale, centralized production systems are the backbone of the economy [1, 2]. While these systems have excelled in mass manufacturing, they rely on long and inflexible supply chains [2] and often inefficiently use limited resources, making them inherently vulnerable [1]. The COVID‐19 pandemic underscored this vulnerability as global supply systems struggled to adapt to sudden shifts in demand and supply, leading to widespread disruptions [1, 3, 4]. Geopolitical events have further exposed the fragility of global supply networks, emphasizing the need for more resilient alternatives [4, 5]. In response, the concept of relocalizing production has gained traction, advocating the use of local resources, labor, and low‐tech solutions to create robust systems [6]. This decentralized approach enhances resilience [1], autonomy, and efficiency while reducing logistical dependencies [4]. A shift toward small‐scale, geographically distributed manufacturing systems is increasingly viewed as a critical step for ensuring sustainability and adaptability.

Distributed manufacturing represents a departure from centralized production systems by utilizing smaller, locally distributed production nodes [1]. It reduces the risk of supply chain disruptions [3], requires less capital and time for production and maintenance, and is particularly accessible in low‐income regions [1]. Moreover, it offers a sustainable alternative by employing decentralized, adaptable, and small‐scale production centers for on‐demand manufacturing, significantly reducing transportation needs and associated emissions [3, 7]. The adoption of distributed and decentralized manufacturing is gaining momentum in emerging markets, driving regional economic growth while promoting sustainable production practices [2, 7]. Beyond production, sustainable development encompasses responsible consumption and the efficient use of natural resources. Distributed manufacturing aligns seamlessly with the principles of sustainable production practices by locating production closer to consumers, fostering both environmental and economic sustainability [7].

The demand for distributed manufacturing using locally accessible sustainable materials was realized during the recent COVID‐19 pandemic for the production of rapid diagnostic tools and personal protective devices [8–11]. The scalable and decentralized manufacturing of low‐cost diagnostic tools for remote analysis is highly anticipated as the global need for quick, accessible solutions continues to rise [11].

In this paper, we demonstrate the production of a paper‐based point‐of‐care (POC) test device to test arsenic contamination in drinking water as an example of distributed manufacturing practice. The arsenic test kit was made on a paper platform, which was made locally from native plant material known as the Lokta plant. In countries like Nepal, the use of Handmade Lokta Paper (HLP) in POC test devices exemplifies the principles of distributed manufacturing. Lokta paper, locally known as Nepali Kagaj, is traditionally crafted from the outer bark of the Lokta plant, such as *Daphne bholua* and *Daphne papyracea* [12]. These plants are naturally regenerative, reaching maturity in 4–5 years, which helps preserve forest ecosystems [13]. HLP is valued for its unique texture, durability, and resilience to insects [8], making it a robust and sustainable material for POC diagnostic devices [14]. Beyond its environmental benefits, HLP also supports local economies by providing income to artisans and preserving traditional manufacturing practices.

We then tested the paper device kit to test arsenic contamination in drinking water. We chose to develop a POC arsenic test device because arsenic is a potent carcinogen affecting approximately 230 million people worldwide. Exposure to arsenic poses significant health risks, including cancers of the bladder, skin, lung, kidney, liver, and prostate [15, 16]. Arsenic contamination in water supplies is primarily caused by industrial effluents, mining activities, and the use of pesticides, where it interacts with oxygen, chemicals, and microorganisms [17–19]. To mitigate the risks, the World Health Organization (WHO) has set the maximum acceptable concentration (MAC) of arsenic in drinking water at 10 ppb [20–25]. In contrast, Nepal’s MAC stands at 50 ppb [15, 25].

Industrial‐scale production of arsenic test kits involves reagent formulation, fabrication of detection strips (drawing, soaking, drying, cutting), and moisture‐proof packaging. Reagents are filled using automated systems, and kits are assembled with vials, reagents, spoons, strips, color charts, instructions, and waste bags. Quality control is conducted using standard arsenic solutions or ICP‐MS, with batch validation for accuracy and shelf‐life. Final steps include labeling with batch codes, expiry dates, safety warnings, and regulatory details. Sustainable, distributed manufacturing can incorporate one or more of these steps. In this study, we aimed to replace commercially manufactured paper with handmade paper derived from locally available plant materials.

To address arsenic‐related problems through affordable testing, arsine‐based gas‐phase colorimetric method is the most common method which is called the Gutzeit method. The inorganic arsenic is converted into arsine gas (AsH_3_) using a reducing agent and detected with the help of subsequent color change [20–22]. This method is popular for its affordability, quick response, and lesser need for professional training, making it suitable for POC testing [22]. However, studies have found instances of false positives and negatives, especially when the concentrations approach 10 µg/L [23]. Further, this reaction uses environmentally concerning mercury‐based reagents. Nonetheless, test kits like Econo‐Quick (EQ) are found to perform correctly making them a good tool for rapid screening [23, 24]. This study chooses the EQ kit as a reference standard against the HLP test strips.

We characterized the properties of HLP and used it to create test strips for arsenic detection in groundwater samples. Key reaction parameters, such as the concentration of mercuric bromide and the dipping time, were optimized to ensure a sustainable and efficient process. The performance of the HLP‐based test strips was then evaluated against a commercial Rapid Arsenic Test Kit for Water Analysis. This approach demonstrates the potential for leveraging locally available materials and distributed manufacturing to create accessible and sustainable diagnostic tools.

## 2. Experimental Section

### 2.1. Materials and Reagents

Potassium peroxomonosulfate (Oxone), zinc dust, and mercuric bromide (HgBr_2_) were bought from Sigma‐Aldrich, Co., India. Sodium hydroxide (NaOH), hydrogen peroxide (H_2_O_2_), citric acid (monohydrate), potassium permanganate (KMnO_4_), and ferrous sulfate (FeSO_4_) were purchased from Thermo Fischer Scientific India Pvt. Ltd., India. Sodium carbonate and sodium bicarbonate were bought from Merck, India. Sodium (meta)arsenite (Na_2_AsO_2_) and sodium arsenate heptahydrate (Na_2_HAsO_4_.7H_2_O) were purchased from Loba Chemicals, India. The raw Lokta fibers were obtained from the Baglung District of Nepal.

### 2.2. Preparation and Characterization of HLP

**FIGURE 1:**
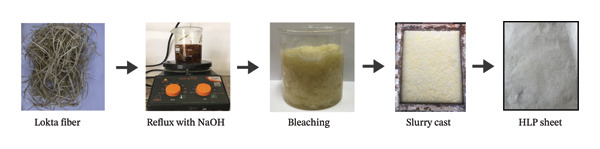
Steps to make Handmade Lokta Paper (HLP) from Lokta fibers.

The outer bark of the Lokta plant was converted into thin, fibrous threads, which were then refluxed in 5% NaOH for 4 hours, cooled, and bleached with a 2% bleaching solution of H_2_O_2_. Then, the pulp was smeared over a screen net submerged in water. The fiber mass was then air‐dried to get the HLP. A schematic of the fabrication process is shown in Figure 1 and has been described previously [26].

(1) moisture content%=initial weight−final weightinitial weight×100.

A high‐precision digital thickness micrometer (SKZ Industrial, China) was used to measure the thickness of the paper sheet, following the TAPPI method [14]. Ten measurements were taken at different places on the paper. For grammage (g/m^2^) measurement, a 100‐cm^2^ piece of paper was cut into a circular shape with a GSM cutter (SKZ Industrial, China), and its weight was measured to the nearest 0.001 g under ambient conditions of temperature and relative humidity, following the TAPPI T410 method. The apparent density was calculated by dividing the grammage by thickness. The equilibrium moisture content was evaluated using the TAPPI T550 test method. The samples were dried, cooled, and weighed three times to obtain the final weight. The following equation was used to calculate the moisture content [27].

The water absorptiveness of circular paper samples was measured using the TAPPI T441 test method. The samples were soaked in 100 mL of water for 60 s in the Cobb‐size tester (Presto, India). The excess water was removed using a blotter paper and a 10 ± 0.5 kg metal roller, and the final weight was taken. For a disc of 100 cm^2^, water absorptiveness was calculated using the following formula [28]:

(2) weight of waterg/m2=final weight–initial weight×100.

The brightness‐opacity tester (SKZ Industrial, China) was used to measure the opacity and brightness (ISO brightness). For every sample, a paper sample of suitable size was cut and put in the sample container.

### 2.3. Preparation of Arsenic Stock Solutions

A 1000  mg/L stock solution of arsenite As(III) was prepared by dissolving 0.173 g of sodium arsenite in deionized (DI) water, and the solution was diluted to 100 mL. Subsequently, this stock solution was subjected to serial dilution to make solutions with lower concentrations (2, 10, 30, 50, 70, 100, 300, 500) µg/L. Similarly, a 1000 mg/L As (V) stock solution was prepared by dissolving 0.416 g of sodium arsenate heptahydrate in DI water, and the total volume was adjusted to 100 mL. Different solutions of lower concentrations were made by serially diluting them.

### 2.4. Fabrication of the Arsenic Paper Strip

Mercuric bromide solutions of concentrations ranging from 0.5% to 6% (w/v) were prepared in absolute ethanol (99.9%). The pieces of HLP with a width of 0.5 cm were dipped in the HgBr_2_ solution of optimized concentration at different times, and these paper pieces were pasted on a plastic sheet using a double‐sided adhesive. The sheet was then cut into multiple strips 0.5 cm wide and 7 cm long.

### 2.5. Arsenic Assessment and Colorimetric Assays

**FIGURE 2:**
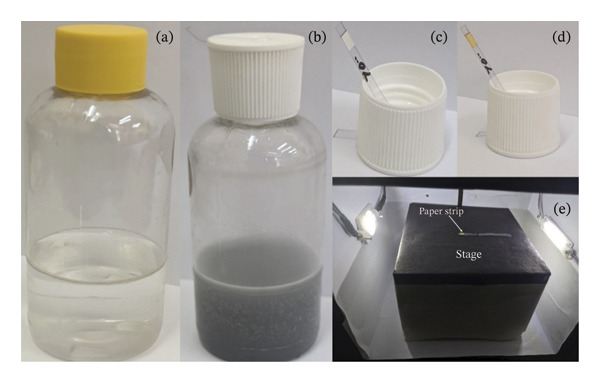
Arsenic assessment method. Arsenic‐containing water sample (a), reaction after addition of reagent and replacement with the turret cap (b), turret cap with test strip—before reaction (c) and after reaction (d), and lightbox with a stage holding the test strip (e).

A 50‐mL aliquot sample was treated with citric acid, ferrous sulfate, and oxone, resulting in a light brownish solution. Finely powdered zinc was added, and the turret cap with the test strip was fixed to the bottle. The evolution of gas bubbles indicated the reaction and the color of the solution changed to ash‐like and gradually colorless. The sensor strip’s detection region turned yellow to brownish due to the formation of mixed mercury‐arsenic halogenide, the color intensity of which depends on the concentration of arsenic in the sample [29]. After 15 min of exposure, the sensor strip was withdrawn and subjected to imaging inside the lightbox (see Figure 2). Triplicate measurements were taken to ensure reproducibility, and the results were compared with the commercial test kit.

Citric acid was used instead of HCl as it is easy to obtain, handle, and transport. This also allows operators to close the reactor vessel lid before excessive arsine gas production, reducing arsine exposure and preventing signal loss. Citric acid reacts with zinc to produce nascent hydrogen, keeping the pH below 2. The nascent hydrogen converts the arsenic into arsine gas and also aids in the upward transportation of arsine, while the oxone removes sulfide interference. The overall reaction mechanisms are represented in the following equations [20, 21]:

(3) 326Zn+C6H8O7⟶Zn3C6H5O72+H

(4) H3AsO3+3633Zn+H+⟶AsH3+Zn2++H2O

(5) AsO43−+41144Zn+H+⟶AsH3+Zn2++H2O

The arsine gas reaches the detection region of the PAD and forms arsenic‐mercury compounds in a stepwise manner [30].

(6) AsH3+HgBr2⟶H2As−HgBr+HBryellow

(7) H2As−HgBr+HgBr2⟶HAsHgBr2+HBrorange

(8) HAsHgBr2+HgBr2⟶AsHgBr3+HBrbrown

### 2.6. Assay Signal Analysis

We assessed the potential of the arsenic assay for qualitative, semiquantitative, and quantitative analyses. Qualitative analysis was performed by visual identification of the presence or absence of arsenic, while semiquantitative analysis was performed by mapping the developed color to the standard color chart. These were rated by eight different raters from different backgrounds. For quantitative analysis, the developed color strips were digitized using a smartphone SM‐E426B and imported to image processing software ImageJ where they were split into separate RGB color spaces and inverted. A custom‐built wooden box with a 2‐cm‐diameter opening at the top and a hinged door was used for taking the image. A 15× 15 cm stage with a black backdrop was used for sample placement and imaging. Two LED lamps were mounted on opposite sides of the white inner wall for constant lighting conditions. The signal was determined by taking the maximum intensity, which is associated with the blue channel [8]. The difference between the acquired signal and the blank assay was used to calculate the net signal. Experiments were conducted at room temperature (∼20°C), during which the relative humidity was ∼40%.

### 2.7. Optimization of Assay Parameters

Mercuric bromide solutions of concentrations (0.5%–6%) were prepared, and rectangular paper pieces were immersed in the solution for 24 h. The paper was then air‐dried and prepared into strips. The strips were then analyzed using 100 µg/L As (III) standard.

The impregnation time of paper pieces is crucial for optimal results after optimizing HgBr_2_ concentration. The paper pieces were immersed in a 1% HgBr_2_ solution for various durations (1 min–30 min) and used to determine arsenic levels in a standard solution.

The exposure time, which is the time required for generating arsine gas and detecting it using paper strips, was also studied and optimized. The paper strips subjected to optimized HgBr_2_ impregnation were exposed to arsine gas generated from the standard for different time durations. Imaging was performed immediately after the withdrawal of the strip from the reaction vessel and analyzed.

Similarly, the paper strips were employed to assess the arsenic standard for 15 min (optimized exposure time) and subjected to imaging at different time intervals from 1 min (immediately) to 35 min to study the trend in delay time for imaging. Triplicate measurements were taken for every experiment to ensure reproducibility.

### 2.8. Groundwater Sample Collection

**FIGURE 3:**
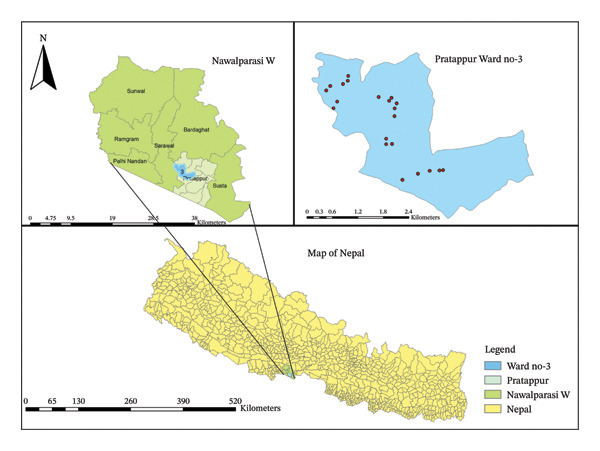
Study area map of Pratappur Rural Municipality Ward 3 in Nawalparasi West district showing sampling sites in red dots.

Arsenic in water has been detected previously in several districts of Nepal. We collected 21 groundwater samples from Pratappur Rural Municipality in Nawalparasi West district of west Nepal on the southern part of the Chure hills. A convenient sampling method was used to collect water samples from handpumps which may serve individual or multiple households for drinking and other household uses. The samples were stored in plastic bottles, transported to the lab, and measured as soon as possible. A map of sampling sites is shown in Figure 3.

## 3. Results and Discussion

We prepared the HLP in the laboratory starting from the bark of the plant and measured several parameters including thickness, grammage, density, and porosity to characterize the paper. These parameters of HLP are found to be comparable to commercial filter papers. Table 1 shows the physical properties of laboratory‐made HLP and commercial filter papers such as Whatman Grade 1 and Grade 4 [31].

**TABLE 1:** Physical properties of Handmade Lokta Paper and commercial Whatman filter papers.

Parameters tested	Lokta paper	Grade 1 [8]	Grade 4 [8]
Thickness (µm)	196.5 ± 49.8	180	205
Grammage (g/m^2^)	76.98	88	96
Apparent density (g/cm^3^)	0.39	0.40	0.46
Apparent porosity (*ε* %)	73.82	68	64
Cobb 60 (g/m^2^)	223.65	NA	NA
Moisture content (%)	3.92 ± 1.21	NA	NA
Brightness (%)	47.34	NA	NA
Opacity (%)	28.82	NA	NA

The thickness of HLP was slightly higher than that of Grade 1 and slightly lower than that of the Grade 4 filter papers. The grammage of HLP was lesser than that of both Whatman filter papers. The apparent density of HLP was comparable to that of Grade 1 Whatman paper, while data showed that HLP is a lightweight paper compared to Grade 4 Whatman filter paper.

The porosity of a paper matrix is the measure of void space that allows air to pass through it. HLP was found to be more porous than the Whatman filter paper. Higher porosity is caused by inadequate calendaring, severe flocculation of fibers, or stiff fibers. Higher porosity provides enhanced solvent absorbency and faster drying [32]. The Cobb 60 value, moisture, brightness, and opacity were found to be 223.65 gm^2^, 3.92 (%), 47.34 (%), and 28.82 (%), respectively.

### 3.1. Optimization of HLP Assay

At first, we optimized the concentration of HgBr_2_ to obtain the best signal for the arsenic assay. We used variable concentrations of the reagent (0.5%–6%) by keeping the concentration of arsenic fixed at 100 µg/L. We found that the signal at 1% (w/v) reagent was highest compared with lower or higher concentrations (Figure 4(a)). It is interesting to note that the optimum reagent concentration is fivefold lower than the reagent used in commercial paper strips. The European Pharmacopoeia specifies that commercial HgBr_2_ PAD manufacturing follows the procedure of impregnation using a 5% HgBr_2_ solution [31, 33], while some studies have used the concentration of up to 6% [30]. The mercuric bromide is highly toxic. Reducing the amount of this reagent for each assay is better in terms of generating less waste and in terms of reducing the exposure to the user.

FIGURE 4Optimizing assay parameters for arsenic detection. (a) Effect of HgBr_2_ concentration on net image intensity. Microscopic images of paper strips with different concentrations of mercuric bromide before and after soaking are provided as inserts. (b) Effect of dipping time on the net signal of image intensity. Assays were performed with 100 µg/L of arsenic with 1% (w/v) HgBr_2_. (c) The net signal varied with the exposure time of the strip in arsenic solution (100 µg/L). (d) Exponential fitting of net image intensity generated by imaging the same paper strip for varying imaging time durations. Error bars are the standard deviations (*n* = 3).

**Figure:**
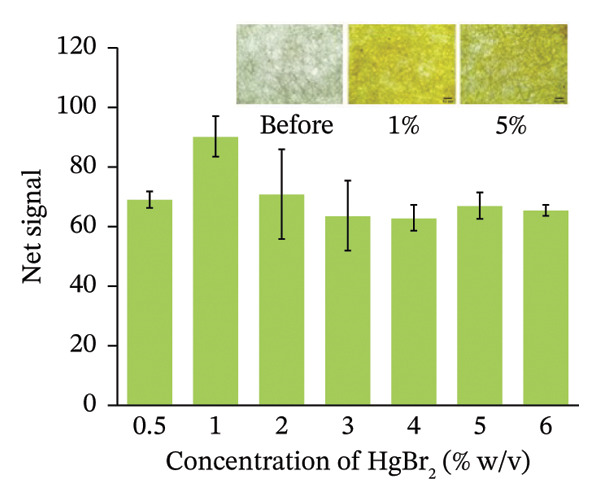
(a)

**Figure:**
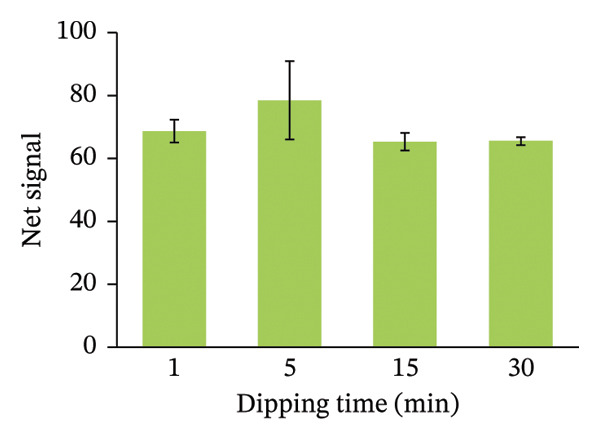
(b)

**Figure:**
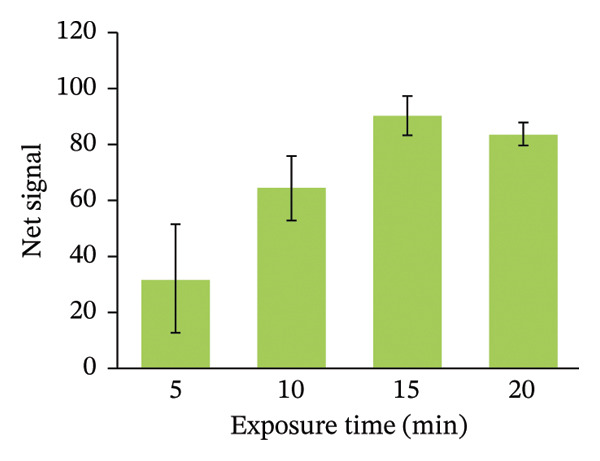
(c)

**Figure:**
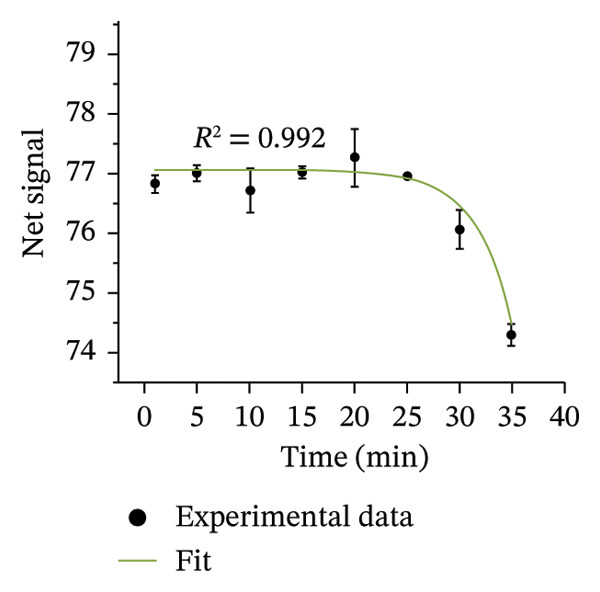
(d)

We also optimized the best time required for the impregnation of HgBr_2_ on the HLP. The paper strips were dipped in the HgBr_2_ solution for different time intervals, and arsenic assays were performed using 100 µg/L of arsenic stock solution with 1% (w/v) HgBr_2._ We found that the highest net signal for the arsenic assay was reached in 5 min (Figure 4(b)). Hence, the dipping time was optimized for 5 min.

Next, the HLP after mercuric bromide impregnation was subjected to detect arsine gas and the exposure time of the arsine gas was optimized. An ideal test should have a shorter exposure time to reduce the assay time but long enough to reduce all the arsenic present in the water sample into arsine gas, which subsequently gets trapped in the HgBr_2_‐impregnated paper strip. We optimized the exposure time by allowing the assay to react for different reaction times, during which the paper strip was allowed to react with the arsine gas for varying time duration and analyzed subsequently. We found that a 15‐min exposure of the paper strip to arsine gas generated from the arsenic solution was enough for the assay (Figure 4(c)). The commercial Kit EQ has recommended 10–12 min of exposure time [34]. Ravula et al. used gold nanoparticles (AuNPs) to detect the arsine gas where they required 2–4 min [20].

After the reaction between arsine gas and mercuric bromide impregnated in the paper, the color is developed on the paper. We recorded the net signal up to 35 min and found that the developed color gradually faded away only after 25 min (Figure 4(d)). Hence, the imaging can be easily performed within 25 min after the paper strip is taken out of the reaction vessel. This optimization also ensures the robustness of the method. The EQ suggests reading the signal before 30 s [34]. This means that the signals should be taken as soon as possible. A robust method should have enough stability so that the results obtained are not much affected by the slight change in environmental conditions and handling of the operator. We optimized the time that an operator can take before imaging the colored strips for quantitative measurement of arsenic concentration.

### 3.2. Qualitative Analysis of Arsenic in Field‐Collected Water Samples

We demonstrated three different methods of analyses of arsenic in the field‐collected water samples—qualitative, semiquantitative, and quantitative methods and compared them with the results from commercial test kit methods.

A qualitative analysis was conducted to screen water quality by detecting the presence or absence of arsenic. This method involved a simple visual inspection of color changes on paper strips. A developed color indicated a positive result, signifying the presence of arsenic, while no color change signifies a negative result or the absence of arsenic. The assessment was performed by comparing the paper strips before and after the reaction.

However, color perception can be subjective, as individuals may interpret the presence, absence, or intensity of color differently. To evaluate this variability, eight students independently analyzed the paper strips qualitatively. Each student was asked to indicate “yes” (presence of color) or “no” (absence of color) for 21 test samples evaluated using both HLP arsenic strips and an EQ kit for comparison. The contingency table summarizing the results from all eight raters is presented in Table 2. Out of 77 total positive responses for the commercial test kit, 1 test response was found to be negative in the HLP arsenic strip method and the rest of them were both positive. This indicated that 1.3% of the test results were false negatives in our test method. Similarly, for the total of 91 negative responses for the EQ kit, 15 responses were found positive in the HLP method.

**TABLE 2:** Contingency table for the visual color detection of test paper strips obtained from both methods. Twenty‐one samples were rated by eight different people.

		Commercial test kit		Row marginals
		Yes	No	
HML strip method	Yes	76	15	91
	No	1	76	77

Column marginals		77	91	168

The results showed that 16.4% of the responses from the HLP strip method were false positives compared to the EQ kit. Various previous reports have noted substantial false‐positive and false‐negative rates of field test kits for arsenic test. For example, a UNICEF report indicates approximately 16% false‐negative results in field testing [35]. A review by Feldmann notes that false‐negative rates exceeding 30% are not unusual for arsenic field kits [36]. Furthermore, a large‐scale study by Rahman et al. reported false negatives as high as 68% and false positives up to 35% [37]. Therefore, these kits shall be used for initial screening purposes with caution [38].

The higher false positive in our case is likely due to the inherent yellow tint of the handmade paper [14], which elevates the background color and affects result interpretation. Both methods identified 10 out of 21 samples as positive. While the relatively high false‐positive rate of the HLP method is a concern, the low incidence of false negatives is encouraging, as it suggests arsenic‐contaminated samples are unlikely to be missed—a critical factor in field testing.

To address the yellow background, bleaching with stronger agents like sodium hypochlorite could be explored. Additionally, digital image processing can help normalize background variations. Techniques such as image thresholding, or converting RGB to HSV or CIELAB color spaces, can improve color accuracy to better align with human visual perception [39]. Incorporating reaction and control zones on the same strip could further minimize misinterpretation by enabling differential analysis against the background. Future work may focus on integrating machine learning (ML) and deep learning (DL) approaches to potentially obtain better results and reduce interpretation errors.

The contingency table shows that most of the results were the same for both test methods. For a few samples, however, the HLP strip method showed the presence of assay color when the commercial kit did not display it.

Most of the raters were using colorimetric test kits for the first time, except one who was experienced and was considered a reference. However, all were master’s‐level chemistry students and were able to follow the instructions with ease. Among the 21 samples tested, 10 contained no arsenic. All raters correctly identified 8 of these 10 samples, while the remaining two were occasionally misclassified as the next higher concentration. Samples in the higher concentration range (30–50 ppm) were accurately identified by all eight raters. Although the lower concentration samples were mostly identified correctly, a few raters misclassified some of them, particularly when using the commercial test kit. The detailed responses are provided in the Supporting Information (Table S2). These results were analyzed by using Cohen’s Kappa statistic to study the degree of agreement between these two test methods.

(9) κ=Pra−Pr  e1−Pr  e.

The formula to calculate Cohen’s Kappa is given by [40].where Pr(*a*) represents the actual observed agreement and Pr(e) represents chance agreement. The values of Pr(*a*) and Pr(*e*) were found to be 0.905 and 0.497, respectively, and the kappa value (*κ*) was calculated to be 0.811. Since the *κ* value was between 0.81 and 1.00, it showed a strong agreement between the two methods [41].

### 3.3. Semiquantitative Analysis of Arsenic in Field‐Collected Water Samples

A semiquantitative analysis of arsenic concentration was conducted by comparing the developed color on the strips with standard color charts (Figure S1). Since color perception varies among individuals, eight participants (*n* = 8) were asked to evaluate the observed color against the standard chart. The samples were categorized into four arsenic concentration ranges: < 10, 10–30, 30–50, and > 50 µg/L.

Among the 21 samples, two were found to have arsenic concentrations exceeding 10 µg/L, with one sample ranging from 10 to 30 µg/L and the other from 30 to 50 µg/L (see Table 3). All other positive samples had arsenic levels below 10 µg/L, which complies with the WHO guideline [25]. The ratings for the semiquantitative analysis were consistent across all participants. Notably, samples S2 and S4 tested positive using the HLP strip method but were negative with the commercial kit, which may be because of the higher sensitivity of our method in detecting lower concentrations, further corroborated by the quantitative analysis presented in the next section.

**TABLE 3:** Results of the semiquantitative assessment of arsenic in 21 water samples according to the standard color charts for each kind of test method.

Sample ID	HLP strip method (μg/L)	EQ kit (μg/L)
S1	—	—
S2	0–10	—
S3	0–10	0–10
S4	0–10	—
S5	0–10	0–10
S6	10–30	10–30
S7	—	—
S8	0–10	0–10
S9	0–10	0–10
S10	0–10	0–10
S11	—	—
S12	0–10	0–10
S13	0–10	0–10
S14	0–10	0–10
S15	30–50	30–50
S16	—	—
S17	—	—
S18	—	—
S19	—	—
S20	—	—
S21	—	—

### 3.4. Quantitative Analysis of Arsenic in Field‐Collected Water Samples

**FIGURE 5:**
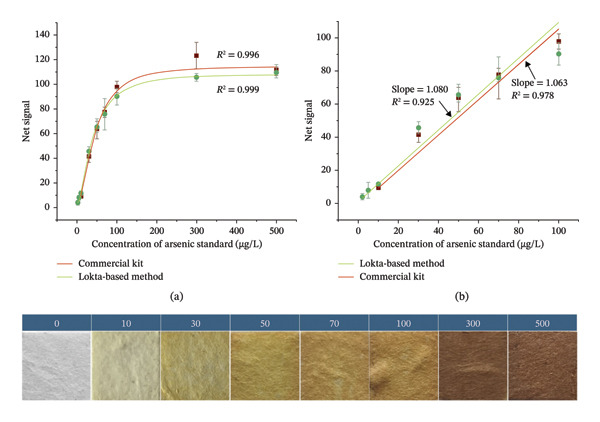
A calibration curve for arsenic assessment with HLP strip method (green) and EQ kit QuickTM (red) (a), linear ranges for both methods (b), standard color chart for semiquantitative analysis (below). Triplicate measurements were taken for each sample.

A response curve was generated using arsenic standard solutions ranging from 2 to 500 µg/L with HLP strips to estimate the limit of detection (LOD) and the limit of quantification (LOQ). For comparison, another response curve was prepared using arsenic standard solutions ranging from 10 to 500 µg/L with EQ kit strips (Figure 5). Blank measurements were conducted using distilled DI water in place of arsenic standards while maintaining identical setups for both methods. Arsenic concentrations for the commercial kit were measured following the manufacturer’s instructions, and imaging was performed similarly. The calibration curves and the slopes of their linear ranges for both methods were compared to evaluate performance.

The coefficient of determination (*R* ^2^ = 0.999) for the HLP strip method was notably higher than that of the EQ kit (*R* ^2^ = 0.996), indicating better overall accuracy. The HLP strip method demonstrated slightly higher sensitivity at lower concentration ranges, while the commercial kit produced higher signals at higher concentrations. Given that naturally occurring arsenic levels in groundwater typically fall around or below 100 µg/L, the HLP strip method showed better performance at lower concentrations. This conclusion is further supported by the slopes of the linear ranges, which indicate sensitivity. The linear detection range for the HLP strip method was 2–100 µg/L, compared to 10–100 µg/L for the EQ kit. The sensitivity slope for the commercial kit was 1.063, while the HLP strip method had a slightly higher slope of 1.080, underscoring its enhanced sensitivity at lower arsenic concentrations.

To calculate LOD and LOQ, 10 blank samples were analyzed, and the mean signal and standard deviation were determined for each method. Using the calculated values in the standard formula, the LOD and LOQ for the EQ kit were found to be 20 and 61 µg/L, respectively, while for the HLP strip method, the LOD and LOQ were slightly lower at 18 and 54 µg/L, respectively. The Pearson correlation coefficient between the calibration curves of the two methods was *r* = +0.9906, with a *p* value < 0.0001. Since *r* > 0.99, this indicates an excellent positive correlation between the two methods, demonstrating similar trends in their calibration performance.

Field samples were analyzed using the optimized conditions for the HLP strip method and the EQ kit. Net signals for each sample were measured using both methods and subsequently fitted to the linear equations derived from their respective calibration curves. The average arsenic concentration in Pratappur Municipality was found to be below 50 µg/L, with groundwater in most locations containing arsenic levels around or below 10 µg/L. The sample with the highest arsenic concentration was from Pidarahani village, ward‐3 (S15). Since the concentrations for most samples were below the LOD, only values exceeding the LOD are reported in Table 4.

**TABLE 4:** Arsenic concentrations in the groundwaters of Pratappur RM—3 measured by the Lokta paper–based method and EQ kit and expressed as µg/L.

Sample ID	HLP strip method (μg/L)	EQ kit (μg/L)
S1	< LOD	< LOD
S2	< LOD	< LOD
S3	< LOD	< LOD
S4	< LOD	< LOD
S5	< LOD	< LOD
S6	26	31
S7	< LOD	< LOD
S8	< LOD	< LOD
S9	< LOD	< LOD
S10	< LOD	< LOD
S11	< LOD	< LOD
S12	< LOD	< LOD
S13	< LOD	< LOD
S14	< LOD	< LOD
S15	42	54
S16	< LOD	< LOD
S17	< LOD	< LOD
S18	< LOD	< LOD
S19	< LOD	< LOD
S20	< LOD	< LOD
S21	< LOD	< LOD

The quantitative assessment of arsenic revealed slightly higher concentrations when using the EQ kit compared to the HLP strip method. This difference can be attributed to the relatively higher background signal observed with the HLP strips, likely due to their yellowish color. Despite this, the results were consistent with the expected range as indicated by the standard color chart. Consequently, the quantitative findings were well aligned with the semiquantitative results.

### 3.5. Cost Estimation

The cost of commercial arsenic test kits currently available in the local market ranges from Rs. 100 to Rs. 750 per test, depending on the brand. For our method, we estimated the cost per test based on direct costs, including materials and chemicals. Among these, mercuric bromide was the most significant expense, while the cost of other chemicals was negligible. However, because a single batch of mercuric bromide solution can be used to dip multiple pieces of paper simultaneously, the production process can be scaled up to thousands of tests without additional material costs, further enhancing the method’s cost‐effectiveness.

Our cost estimation, which excludes plastic bottles and labor costs, indicates a direct cost of Rs. 50 per test for our method. While this calculation does not include indirect costs, the significantly lower price compared to EQ kits highlights the potential of our approach as a low‐cost arsenic diagnostic tool. A detailed breakdown of the cost calculation is provided in the Supporting Information (Table S1), demonstrating the scalability and affordability of this method for widespread application.

## 4. Conclusions

We demonstrated the potential of HLP as a sustainable and cost‐effective substrate for decentralized manufacturing of POC arsenic detection devices. By utilizing native plant materials from western Nepal and optimizing the paper‐making process, we achieved a significant reduction in the use of toxic chemicals and dependence on centralized supply chains. The HLP‐based paper strip sensor exhibited excellent analytical performance, including lower LOD (18 µg/L) and LOQ (54 µg/L) and strong agreement with the EQkit. The integration of traditional materials like Lokta paper into advanced analytical applications exemplifies a model for sustainable innovation. Our approach not only addresses pressing global health challenges, such as arsenic contamination in drinking water, but also fosters local economic development and environmental resilience. The scalable, low‐cost production of these devices offers a viable pathway to improve water safety in resource‐limited settings while empowering local communities through the preservation of traditional craftsmanship and employment opportunities. However, a significant effort must be made on quality control to maintain consistent test performance by training the local producers and conducting further research. Future work may explore expanding the application of Lokta paper in other sensing tools, enhancing its properties through material engineering, and validating its efficacy across diverse environmental and clinical scenarios. By leveraging sustainable practices and decentralized manufacturing, this work paves the way for broader adoption of environmentally friendly technologies in addressing global challenges.

## Funding

This work was supported by the Collaborative Research and Innovative Grant (CRIG‐78/79‐S&T‐02) from the University Grants Commission (UGC), Nepal

## Disclosure

A preprint of this manuscript has previously been published [42].

## Conflicts of Interest

The authors declare no conflicts of interest.

## Supporting Information

Additional supporting information can be found online in the Supporting Information section.

## Supporting information


**Supporting Information** Figure S1: Standard color chart used for qualitative and semiquantitative arsenic assessment—for commercial test kit (above) and for HLP method (below). Table S1: The table presents the estimated cost per test for the arsenic assay using Handmade Lokta Paper (HLP) strip, considering only the expenses for chemicals and materials used. It excludes labor cost. The primary purpose of this estimation is to provide a general overview of the cost‐efficiency of the HLP‐based method.

## Data Availability

The data that support the findings of this study are provided in Supporting Information.
