# Supplementary material for: Affordable Arsenic Detection Device Using Handmade Lokta Paper for Decentralized and Sustainable Manufacturing
Source: J Anal Methods Chem. 2026 May 23;2026:9918530. doi: 10.1155/jamc/9918530 (PMC13197832; doi:10.1155/jamc/9918530)
Supplement: Supplementary file 1 — Supporting Information Figure S1: Standard color chart used for qualitative and semiquantitative arsenic assessment—for commercial test kit (above) and for HLP method (below). Table S1: The table presents the estimated cost per test for the arsenic assay using Handmade Lokta Paper (HLP) strip, considering only the expenses for chemicals and materials used. It excludes labor cost. The primary purpose of this estimation is to provide a general overview of the cost‐efficiency of the HLP‐based method. [file JAMC-2026-9918530-s001.docx]

# Supplementary Information

*for*

# Affordable Arsenic Detection Devices Using Handmade Lokta Paper for Decentralized and Sustainable Manufacturing

Ramdeep Shrestha^1,2^, Bhanu Bhakta Neupane^1^*, Basant Giri^2^*

^1^Central Department of Chemistry, Tribhuvan University, Kirtipur, Nepal

^2^Center for Analytical Sciences, Kathmandu Institute of Applied Sciences, PO Box 23003 Kathmandu, Nepal

*Correspondence: bhanu.neupane@cdc.tu.edu.np/ bgiri@kias.org.np


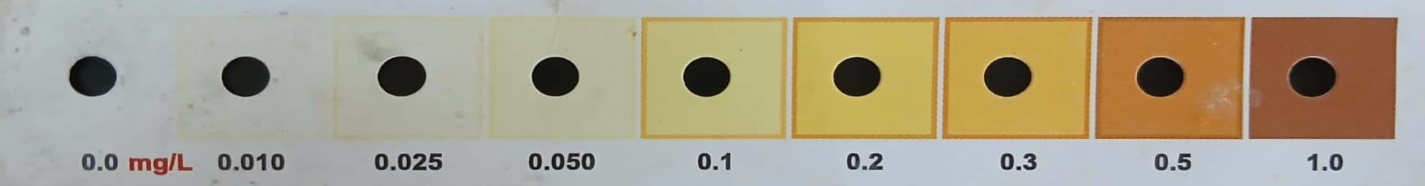


**Figure S1**: Standard color chart used for qualitative and semi-quantitative arsenic assessment – for commercial test kit (above), and for HLP method (below).

## Cost estimation per test

Table S1: The table presents the estimated cost per test for the arsenic assay using Handmade Lokta Paper (HLP) strip, considering only the expenses for chemicals and materials used. It excludes labor cost. The primary purpose of this estimation is to provide a general overview of the cost-efficiency of the HLP-based method.

| **materials** | **Qty** | **Rate (NPR)** | **Qty per tests** | **Price per test** |
| --- | --- | --- | --- | --- |
| ferrous sulfate | 500g | 640 | 0.12 mg | 0.0001 |
| zinc dust | 500g | 2770 | 0.3 mg | 0.002 |
| citric acid | 500g | 1245 | 1.2 mg | 0.003 |
| Lokta paper | A4 (2478 pieces (0.5*0.5 cm2) | 11 | 1pc | 0.004 |
| potassium peroxomonosulfate | 100g | 8490 | 0.12 mg | 0.01 |
| plastic sheet | A4 (177 pieces (7*0.5 cm2) | 5 | 1pc | 0.03 |
| mercuric bromide | 100g | 19600 | 0.25g | 49 |
|  |  |  | **Total (NPR)** | **49.05** |

## Calculation of LOD and LOQ

Limit of Detection (LOD) is calculated by using the following formula.

|  | $LOD=3.3\frac{s}{S}$ |  |
| --- | --- | --- |

Similarly, the Limit of Quantification (LOQ) was calculated using the formula below.

|  | $LOQ=10\frac{s}{S}$ |  |
| --- | --- | --- |

Where, s is the standard deviation of blank measurements and S is the slope of the calibration curve.

### For the commercial kit method

- S = 6.435 and S = 1.063
- LOD = 19.9 ppb
- LOQ = 60.5ppb

### For the HLP-based method

- S = 5.824 and S = 1.080
- LOD = 17.8 ppb
- LOQ = 53.9 ppb
